# 2-Isopropyl-5-methyl­cyclo­hexyl 5-acet­oxy-1,3-oxathiol­ane-2-carboxyl­ate

**DOI:** 10.1107/S1600536809046492

**Published:** 2009-11-21

**Authors:** Grzegorz Dutkiewicz, C. S. Chidan Kumar, H. S. Yathirajan, A. N. Mayekar, Maciej Kubicki

**Affiliations:** a Department of Chemistry, Adam Mickiewicz University, Grunwaldzka 6, 60-780 Poznań, Poland; b Department of Studies in Chemistry, University of Mysore, Manasagangotri, Mysore 570 006, India; c Sequent Scientific Limited, New Mangalore 575 011, India

## Abstract

In the title compound, C_16_H_26_O_5_S, the oxathiol­ane ring adopts an envelope conformation, with the S atom 0.793 (3) Å out of the mean plane of the remaining four atoms. The cyclo­hexane ring of the menthol fragment adopts an almost ideal chair conformation, with all substituents in the equatorial positions. In the crystal, relatively strong, short and linear C—H⋯O hydrogen bonds link the mol­ecules into the chains along [100] direction. The chains are packed into the crystal structure by means of weak dispersive inter­actions. Inter­molecular C—H⋯S inter­actions are also observed.

## Related literature

The title compound is a drug inter­mediate of lamivudine, a reverse transcriptase inhibitor used in the treatment of HIV infections. For the structures of lamivudine and its hydrate have been studied, see: Harris *et al.* (1997 ▶). For the identification of lamivudine conformers by Raman scattering measurements and quantum chemical calculations, see: Pereira *et al.* (2007 ▶). For asymmetry parameters, see: Duax & Norton (1975 ▶). For a description of the Cambridge Structural Database, see: Allen (2002 ▶).
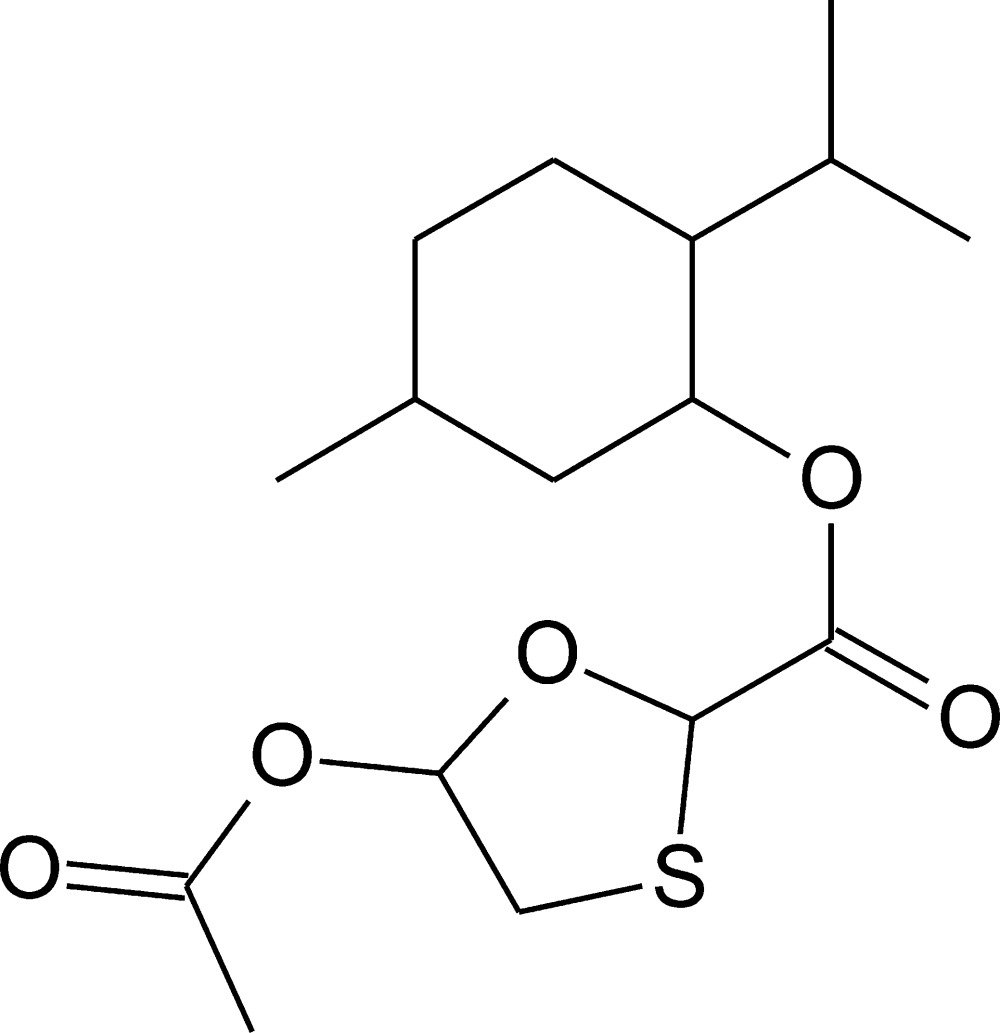


## Experimental

### 

#### Crystal data

C_16_H_26_O_5_S
*M* *_r_* = 330.43
Orthorhombic, 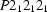

*a* = 5.329 (1) Å
*b* = 13.867 (1) Å
*c* = 23.490 (2) Å
*V* = 1735.8 (4) Å^3^
*Z* = 4
Mo *K*α radiation
μ = 0.21 mm^−1^
*T* = 100 K
0.3 × 0.3 × 0.15 mm

#### Data collection

Oxford Diffraction Xcalibur Sapphire2 large Be window diffractometer
Absorption correction: multi-scan (*CrysAlis Pro*; Oxford Diffraction, 2009 ▶) *T*_min_ = 0.719, *T*_max_ = 1.000
11503 measured reflections
3632 independent reflections
3175 reflections with *I* > 2σ(*I*)
*R*_int_ = 0.025

#### Refinement

*R*[*F* ^2^ > 2σ(*F* ^2^)] = 0.028
*wR*(*F* ^2^) = 0.053
*S* = 1.03
3632 reflections
277 parameters
H atoms treated by a mixture of independent and constrained refinement
Δρ_max_ = 0.28 e Å^−3^
Δρ_min_ = −0.18 e Å^−3^
Absolute structure: Flack (1983 ▶), 1435 Friedel pairs
Flack parameter: −0.04 (5)

### 

Data collection: *CrysAlis Pro* (Oxford Diffraction, 2009 ▶); cell refinement: *CrysAlis Pro*; data reduction: *CrysAlis Pro*; program(s) used to solve structure: *SIR92* (Altomare *et al.*, 1993 ▶); program(s) used to refine structure: *SHELXL97* (Sheldrick, 2008 ▶); molecular graphics: *Stereochemical Workstation Operation Manual* (Siemens, 1989 ▶); software used to prepare material for publication: *SHELXL97*.

## Supplementary Material

Crystal structure: contains datablocks I, global. DOI: 10.1107/S1600536809046492/jh2112sup1.cif

Structure factors: contains datablocks I. DOI: 10.1107/S1600536809046492/jh2112Isup2.hkl

Additional supplementary materials:  crystallographic information; 3D view; checkCIF report

## Figures and Tables

**Table 1:** Hydrogen-bond geometry (Å, °)

*D*—H⋯*A*	*D*—H	H⋯*A*	*D*⋯*A*	*D*—H⋯*A*
C13—H13⋯O12^i^	0.989 (15)	2.261 (15)	3.1563 (18)	150.0 (12)
C15—H15*A*⋯S14^ii^	0.961 (16)	3.033 (15)	3.7794 (15)	135.6 (11)
C20—H20*B*⋯O19^i^	0.961 (18)	2.524 (18)	3.464 (2)	166.0 (14)

Symmetry codes: (i) 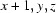; (ii) 
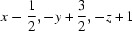
.

**Fig. 1.**
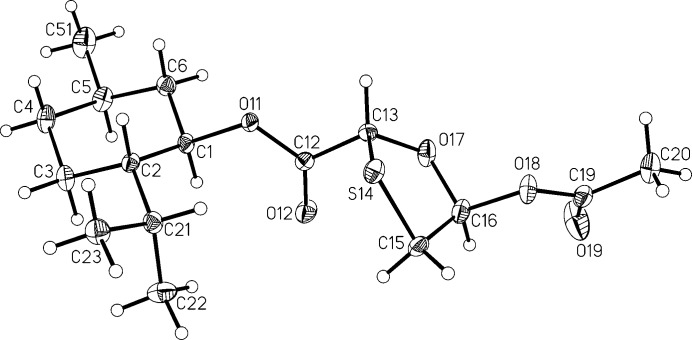
Anisotropic ellipsoid representation of the compound I together with atom labelling scheme. The ellipsoids are drawn at 50% probability level, hydrogen atoms are depicted as spheres with arbitrary radii.

**Fig. 2.**
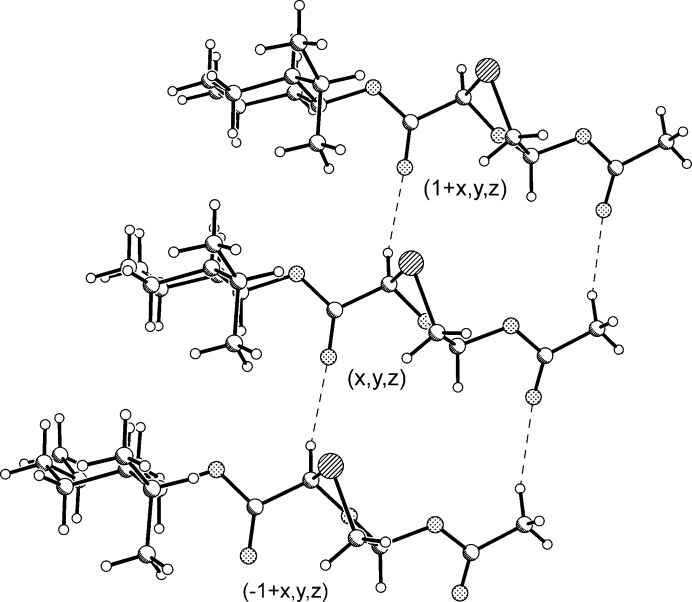
The hydrogen-bonded chain of molecules of I. Hydrogen bonds are shown as dashed lines.

**Fig. 3.**
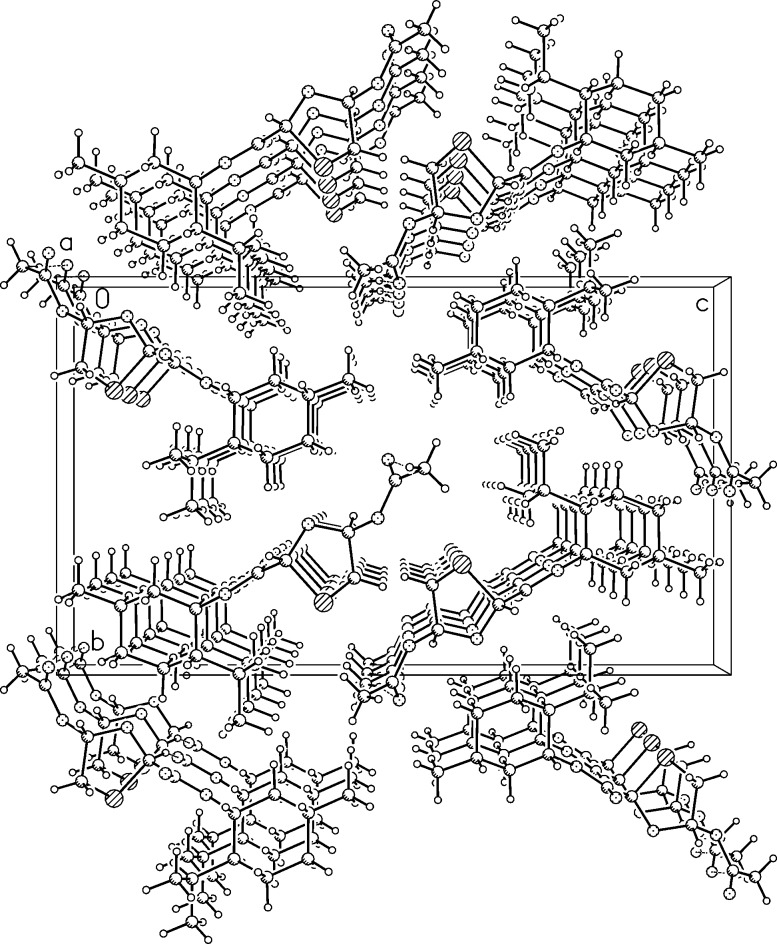
The crystal packing as seen along the chain direction, i.e. along [100].

**Fig. 4.**
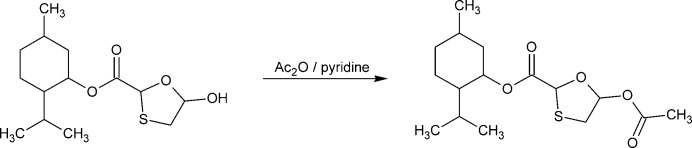
The preparation of the title compound.

## supplementary crystallographic information

### Comment

5-Methyl-2-(propan-2-yl)cyclohexyl 5-(acetyloxy)-1,3-oxathiolane-2-carboxylate (I, Scheme 1) is a drug intermediate of lamivudine which is a reverse transcriptase inhibitor used in the treatment of HIV infection alone or in combination with other class of Anti HIV drugs. The crystal stractures of Lamivudine and its hydrate have been studied (Harris *et al.*, 1997). The identification of lamivudine conformers by Raman scattering measurements and quantum chemical calculations is reported (Pereira *et al.*, 2007).

The conformation of the oxathiolane ring is close to an envelope (Fig. 1), with four atoms C13, C15, C16 and O17 almost coplanar (maximum deviation from the least-squares plane of 0.0469 (11) Å) while the fifth atom (S14) is significantly, by 0.793 (3) Å out of this plane. Also the asymmetry parameter (Duax & Norton, 1975), which describes the deviation from the ideal symmetry (in this case *C_s_*), has relatively low value of 6.0°. Similar conformation was observed in the majority of the structures with not fused oxathiolane rings found in the Cambridge Structural Database (Allen, 2002), however different atoms occupy the out-of-plane position. The acetyloxy substituent occupies the *quasi*-axial position with respect to the oxathiolane ring (C13—O17—C16—O18 torsion angle is -109.18 (13) °, S14—C15—C16—O18 84.13 (12) °), the position of carboxylate group is also close to the axial one (C16—O17—C13—C12 - 97.66 (14) °, C15—S14—C13—C12 84.89 (10) °). The cyclohexyl ring is close to the typical chair conformation (maximum and minimum values of the asymmetry parameters are 0.74° for ΔC_s_^5^, and 3.96° ΔC_2_^1–6^), all substituents: methyl, isopropyl and carboxylate are in equatorial positions.

In the crystal structure relatively strong (short and directional) C13—H13···O12^i^ hydrogen bonds join the molecules into the infinite chains along [100]. These chain in turn are organized into the crystal structure by weak van der Waals - type interactions (Table 1, Fig. 2).

### Experimental

To a mixture of *L*-menthyl-5-hydroxy-1,3-oxathiolane-2-carboxylate (1.5 g, 5.2 m mol) in pyridine (30 ml), acetic anhydride (6.4 ml) was added slowly at 273 K (Fig. 4). The mixture was allowed to attain room temperature and stirred over night, then quenched to ice cold water and extracted with ethyl acetate. The organic layer was concentrated under vacuum to obtain the product. X-ray quality crystals were grown from slow evaporation of methanol solution (m.p.: 333–335 K).

### Refinement

Positional parameters of the hydrogen atoms were freely refined, the *U*_iso_ values of these atoms were set at 1.2 (1.5 for methyl groups) times *U*_eq_ of their carrier carbon atom.

### Crystal data

**Table:**

C_16_H_26_O_5_S	*F*(000) = 712
*M**_r_* = 330.43	*D*_x_ = 1.264 Mg m^−^^3^
Orthorhombic, *P*2_1_2_1_2_1_	Mo *K*α radiation, λ = 0.71073 Å
Hall symbol: P 2ac 2ab	Cell parameters from 3953 reflections
*a* = 5.329 (1) Å	θ = 3.0–75.3°
*b* = 13.867 (1) Å	µ = 0.21 mm^−^^1^
*c* = 23.490 (2) Å	*T* = 100 K
*V* = 1735.8 (4) Å^3^	Prism, colourless
*Z* = 4	0.3 × 0.3 × 0.15 mm

### Data collection

**Table:**

Oxford Diffraction Xcalibur Sapphire2 large Be window diffractometer	3632 independent reflections
Radiation source: Nova (Mo) X-ray Source	3175 reflections with *I* > 2σ(*I*)
graphite	*R*_int_ = 0.025
Detector resolution: 5.2679 pixels mm^-1^	θ_max_ = 27.8°, θ_min_ = 2.9°
ω scans	*h* = −6→6
Absorption correction: multi-scan (*CrysAlis PRO*; Oxford Diffraction, 2009)	*k* = −17→19
*T*_min_ = 0.719, *T*_max_ = 1.000	*l* = −18→28
11503 measured reflections	

### Refinement

**Table:**

Refinement on *F*^2^	Secondary atom site location: difference Fourier map
Least-squares matrix: full	Hydrogen site location: inferred from neighbouring sites
*R*[*F*^2^ > 2σ(*F*^2^)] = 0.028	H atoms treated by a mixture of independent and constrained refinement
*wR*(*F*^2^) = 0.053	*w* = 1/[σ^2^(*F*_o_^2^) + (0.026*P*)^2^] where *P* = (*F*_o_^2^ + 2*F*_c_^2^)/3
*S* = 1.03	(Δ/σ)_max_ = 0.001
3632 reflections	Δρ_max_ = 0.28 e Å^−^^3^
277 parameters	Δρ_min_ = −0.18 e Å^−^^3^
0 restraints	Absolute structure: Flack (1983), 1435 Friedel pairs
Primary atom site location: structure-invariant direct methods	Flack parameter: −0.04 (5)

### Special details

**Table:**

Geometry. All e.s.d.'s (except the e.s.d. in the dihedral angle between two l.s. planes) are estimated using the full covariance matrix. The cell e.s.d.'s are taken into account individually in the estimation of e.s.d.'s in distances, angles and torsion angles; correlations between e.s.d.'s in cell parameters are only used when they are defined by crystal symmetry. An approximate (isotropic) treatment of cell e.s.d.'s is used for estimating e.s.d.'s involving l.s. planes.
Refinement. Refinement of *F*^2^ against ALL reflections. The weighted *R*-factor *wR* and goodness of fit *S* are based on *F*^2^, conventional *R*-factors *R* are based on *F*, with *F* set to zero for negative *F*^2^. The threshold expression of *F*^2^ > σ(*F*^2^) is used only for calculating *R*-factors(gt) *etc*. and is not relevant to the choice of reflections for refinement. *R*-factors based on *F*^2^ are statistically about twice as large as those based on *F*, and *R*- factors based on ALL data will be even larger.

### Fractional atomic coordinates and isotropic or equivalent isotropic displacement parameters (Å^2^)

**Table:**

	*x*	*y*	*z*	*U*_iso_*/*U*_eq_	
C1	0.2221 (3)	0.81153 (11)	0.22603 (6)	0.0144 (3)	
H1	0.055 (3)	0.7890 (12)	0.2363 (5)	0.017*	
C2	0.2271 (3)	0.92125 (11)	0.22689 (6)	0.0133 (3)	
H2	0.402 (3)	0.9383 (11)	0.2176 (6)	0.016*	
C21	0.1670 (3)	0.96521 (11)	0.28540 (6)	0.0163 (3)	
H21	0.277 (3)	0.9324 (11)	0.3133 (7)	0.020*	
C22	−0.1041 (3)	0.95024 (13)	0.30376 (8)	0.0248 (4)	
H22A	−0.139 (3)	0.9755 (13)	0.3442 (8)	0.037*	
H22B	−0.154 (3)	0.8837 (14)	0.3016 (7)	0.037*	
H22C	−0.216 (3)	0.9878 (14)	0.2804 (8)	0.037*	
C23	0.2308 (3)	1.07241 (12)	0.28675 (7)	0.0207 (4)	
H23A	0.126 (3)	1.1091 (13)	0.2621 (7)	0.031*	
H23B	0.396 (3)	1.0834 (12)	0.2739 (7)	0.031*	
H23C	0.207 (3)	1.0990 (12)	0.3264 (7)	0.031*	
C3	0.0559 (3)	0.95554 (11)	0.17848 (6)	0.0193 (3)	
H3A	−0.122 (3)	0.9338 (12)	0.1888 (7)	0.023*	
H3B	0.049 (3)	1.0270 (12)	0.1771 (6)	0.023*	
C4	0.1377 (3)	0.91614 (12)	0.12123 (7)	0.0222 (4)	
H4A	0.300 (3)	0.9392 (12)	0.1134 (7)	0.027*	
H4B	0.025 (3)	0.9406 (12)	0.0913 (7)	0.027*	
C5	0.1436 (3)	0.80595 (12)	0.12053 (6)	0.0199 (4)	
H5	−0.025 (3)	0.7843 (12)	0.1282 (6)	0.024*	
C51	0.2389 (4)	0.76739 (13)	0.06413 (8)	0.0305 (4)	
H51A	0.398 (4)	0.7932 (14)	0.0557 (7)	0.046*	
H51B	0.137 (3)	0.7914 (15)	0.0335 (8)	0.046*	
H51C	0.245 (3)	0.6975 (14)	0.0650 (8)	0.046*	
C6	0.3052 (3)	0.76948 (11)	0.16982 (6)	0.0172 (3)	
H6A	0.478 (3)	0.7877 (12)	0.1612 (6)	0.021*	
H6B	0.305 (3)	0.6970 (12)	0.1714 (6)	0.021*	
O11	0.39944 (16)	0.77425 (7)	0.26904 (4)	0.0146 (2)	
C12	0.3058 (3)	0.72616 (10)	0.31299 (6)	0.0134 (3)	
O12	0.08816 (18)	0.70999 (9)	0.32180 (4)	0.0214 (2)	
C13	0.5134 (3)	0.69492 (11)	0.35302 (6)	0.0146 (3)	
H13	0.672 (3)	0.6830 (11)	0.3325 (6)	0.018*	
S14	0.56357 (7)	0.79072 (3)	0.404699 (15)	0.01686 (9)	
C15	0.3109 (3)	0.73820 (11)	0.44548 (6)	0.0176 (3)	
H15A	0.327 (3)	0.7581 (11)	0.4845 (7)	0.021*	
H15B	0.150 (3)	0.7576 (11)	0.4298 (7)	0.021*	
C16	0.3439 (3)	0.63111 (12)	0.43767 (6)	0.0189 (3)	
H16	0.175 (3)	0.5948 (11)	0.4441 (6)	0.023*	
O17	0.4379 (2)	0.61176 (7)	0.38320 (4)	0.0205 (2)	
O18	0.52454 (18)	0.59729 (7)	0.47832 (4)	0.0206 (2)	
C19	0.4838 (3)	0.50989 (11)	0.50215 (6)	0.0193 (3)	
O19	0.3034 (2)	0.46223 (9)	0.49229 (6)	0.0418 (4)	
C20	0.6923 (3)	0.48274 (13)	0.54115 (7)	0.0252 (4)	
H20A	0.709 (3)	0.5287 (14)	0.5686 (8)	0.038*	
H20B	0.850 (3)	0.4807 (13)	0.5212 (8)	0.038*	
H20C	0.659 (3)	0.4227 (13)	0.5576 (7)	0.038*	

### Atomic displacement parameters (Å^2^)

**Table:**

	*U*^11^	*U*^22^	*U*^33^	*U*^12^	*U*^13^	*U*^23^
C1	0.0124 (7)	0.0178 (9)	0.0129 (8)	−0.0003 (7)	−0.0033 (6)	0.0033 (6)
C2	0.0115 (7)	0.0157 (8)	0.0127 (8)	−0.0026 (6)	−0.0008 (6)	0.0021 (6)
C21	0.0179 (8)	0.0171 (8)	0.0137 (8)	0.0017 (7)	−0.0020 (6)	0.0002 (6)
C22	0.0232 (9)	0.0248 (10)	0.0265 (10)	−0.0013 (8)	0.0080 (8)	−0.0066 (7)
C23	0.0247 (9)	0.0181 (9)	0.0192 (10)	0.0005 (7)	−0.0012 (7)	−0.0027 (7)
C3	0.0246 (9)	0.0149 (8)	0.0184 (8)	0.0007 (8)	−0.0050 (7)	0.0025 (6)
C4	0.0310 (10)	0.0200 (9)	0.0157 (8)	−0.0019 (7)	−0.0068 (7)	0.0038 (7)
C5	0.0255 (8)	0.0188 (9)	0.0155 (8)	−0.0053 (7)	−0.0037 (6)	−0.0011 (7)
C51	0.0474 (12)	0.0260 (11)	0.0180 (9)	−0.0037 (10)	−0.0045 (8)	−0.0035 (8)
C6	0.0198 (8)	0.0142 (9)	0.0175 (8)	0.0005 (7)	0.0004 (6)	−0.0005 (6)
O11	0.0124 (5)	0.0178 (6)	0.0137 (5)	0.0008 (5)	−0.0006 (4)	0.0037 (4)
C12	0.0159 (8)	0.0115 (8)	0.0126 (8)	−0.0005 (6)	0.0010 (6)	−0.0021 (6)
O12	0.0138 (5)	0.0287 (6)	0.0218 (6)	−0.0059 (5)	−0.0009 (4)	0.0072 (5)
C13	0.0158 (8)	0.0156 (8)	0.0124 (7)	0.0031 (6)	0.0013 (5)	−0.0005 (6)
S14	0.01923 (18)	0.01732 (18)	0.01403 (18)	−0.00361 (17)	−0.00206 (15)	0.00048 (16)
C15	0.0176 (8)	0.0250 (9)	0.0101 (8)	0.0018 (7)	0.0015 (6)	0.0009 (6)
C16	0.0186 (8)	0.0236 (9)	0.0145 (8)	−0.0040 (8)	−0.0029 (6)	0.0058 (7)
O17	0.0326 (6)	0.0136 (5)	0.0153 (5)	0.0006 (5)	−0.0039 (5)	0.0012 (4)
O18	0.0229 (6)	0.0205 (6)	0.0183 (5)	−0.0065 (5)	−0.0073 (4)	0.0079 (4)
C19	0.0224 (8)	0.0180 (8)	0.0174 (8)	−0.0002 (7)	0.0028 (6)	0.0046 (6)
O19	0.0291 (6)	0.0330 (8)	0.0632 (9)	−0.0137 (6)	−0.0157 (6)	0.0248 (7)
C20	0.0335 (10)	0.0230 (10)	0.0193 (9)	−0.0006 (8)	−0.0049 (8)	0.0050 (8)

### Geometric parameters (Å, °)

**Table:**

C1—O11	1.4768 (16)	C51—H51A	0.94 (2)
C1—C6	1.510 (2)	C51—H51B	0.961 (18)
C1—C2	1.522 (2)	C51—H51C	0.970 (19)
C1—H1	0.975 (15)	C6—H6A	0.975 (15)
C2—C3	1.533 (2)	C6—H6B	1.006 (16)
C2—C21	1.537 (2)	O11—C12	1.3264 (16)
C2—H2	0.986 (15)	C12—O12	1.1991 (16)
C21—C22	1.522 (2)	C12—C13	1.5155 (19)
C21—C23	1.525 (2)	C13—O17	1.4122 (17)
C21—H21	0.990 (16)	C13—S14	1.8193 (15)
C22—H22A	1.029 (19)	C13—H13	0.989 (15)
C22—H22B	0.961 (19)	S14—C15	1.8059 (16)
C22—H22C	0.965 (18)	C15—C16	1.507 (2)
C23—H23A	0.951 (17)	C15—H15A	0.961 (16)
C23—H23B	0.945 (18)	C15—H15B	0.973 (15)
C23—H23C	1.009 (17)	C16—O17	1.4000 (18)
C3—C4	1.515 (2)	C16—O18	1.4345 (17)
C3—H3A	1.024 (16)	C16—H16	1.042 (16)
C3—H3B	0.992 (16)	O18—C19	1.3526 (17)
C4—C5	1.528 (2)	C19—O19	1.1891 (17)
C4—H4A	0.941 (18)	C19—C20	1.489 (2)
C4—H4B	0.983 (16)	C20—H20A	0.911 (19)
C5—C51	1.516 (2)	C20—H20B	0.961 (18)
C5—C6	1.529 (2)	C20—H20C	0.936 (18)
C5—H5	0.965 (15)		
			
O11—C1—C6	106.00 (11)	C6—C5—H5	106.3 (9)
O11—C1—C2	109.26 (12)	C5—C51—H51A	110.6 (11)
C6—C1—C2	113.12 (13)	C5—C51—H51B	109.9 (11)
O11—C1—H1	107.7 (8)	H51A—C51—H51B	102.8 (15)
C6—C1—H1	111.1 (8)	C5—C51—H51C	110.2 (11)
C2—C1—H1	109.5 (10)	H51A—C51—H51C	110.8 (17)
C1—C2—C3	106.85 (12)	H51B—C51—H51C	112.3 (17)
C1—C2—C21	113.86 (13)	C1—C6—C5	111.69 (12)
C3—C2—C21	114.59 (13)	C1—C6—H6A	111.0 (9)
C1—C2—H2	104.7 (9)	C5—C6—H6A	106.8 (9)
C3—C2—H2	108.9 (8)	C1—C6—H6B	110.7 (9)
C21—C2—H2	107.4 (8)	C5—C6—H6B	111.0 (9)
C22—C21—C23	109.79 (14)	H6A—C6—H6B	105.5 (13)
C22—C21—C2	113.39 (13)	C12—O11—C1	117.88 (11)
C23—C21—C2	111.01 (13)	O12—C12—O11	126.32 (13)
C22—C21—H21	108.3 (9)	O12—C12—C13	123.07 (13)
C23—C21—H21	107.6 (9)	O11—C12—C13	110.60 (11)
C2—C21—H21	106.5 (9)	O17—C13—C12	109.67 (12)
C21—C22—H22A	112.7 (9)	O17—C13—S14	107.65 (9)
C21—C22—H22B	112.2 (10)	C12—C13—S14	108.21 (10)
H22A—C22—H22B	109.0 (15)	O17—C13—H13	110.7 (9)
C21—C22—H22C	110.8 (11)	C12—C13—H13	111.7 (9)
H22A—C22—H22C	103.2 (14)	S14—C13—H13	108.8 (9)
H22B—C22—H22C	108.5 (15)	C15—S14—C13	87.13 (7)
C21—C23—H23A	112.2 (10)	C16—C15—S14	104.23 (11)
C21—C23—H23B	111.0 (11)	C16—C15—H15A	112.8 (9)
H23A—C23—H23B	105.6 (14)	S14—C15—H15A	108.9 (9)
C21—C23—H23C	110.4 (10)	C16—C15—H15B	109.2 (9)
H23A—C23—H23C	107.0 (14)	S14—C15—H15B	110.3 (9)
H23B—C23—H23C	110.5 (14)	H15A—C15—H15B	111.1 (13)
C4—C3—C2	112.04 (14)	O17—C16—O18	107.80 (12)
C4—C3—H3A	111.7 (9)	O17—C16—C15	109.99 (12)
C2—C3—H3A	106.5 (9)	O18—C16—C15	108.64 (12)
C4—C3—H3B	110.0 (8)	O17—C16—H16	110.4 (8)
C2—C3—H3B	110.9 (9)	O18—C16—H16	109.0 (8)
H3A—C3—H3B	105.5 (13)	C15—C16—H16	111.0 (8)
C3—C4—C5	112.08 (13)	C16—O17—C13	113.85 (11)
C3—C4—H4A	108.4 (10)	C19—O18—C16	117.42 (11)
C5—C4—H4A	108.6 (10)	O19—C19—O18	123.18 (13)
C3—C4—H4B	109.6 (9)	O19—C19—C20	125.64 (15)
C5—C4—H4B	110.5 (9)	O18—C19—C20	111.18 (13)
H4A—C4—H4B	107.6 (13)	C19—C20—H20A	109.4 (11)
C51—C5—C4	111.65 (14)	C19—C20—H20B	111.1 (10)
C51—C5—C6	110.89 (13)	H20A—C20—H20B	106.0 (16)
C4—C5—C6	109.53 (13)	C19—C20—H20C	109.6 (11)
C51—C5—H5	111.4 (9)	H20A—C20—H20C	110.5 (16)
C4—C5—H5	106.9 (10)	H20B—C20—H20C	110.1 (15)
			
O11—C1—C2—C3	175.68 (11)	C1—O11—C12—O12	−0.5 (2)
C6—C1—C2—C3	57.86 (16)	C1—O11—C12—C13	−179.08 (11)
O11—C1—C2—C21	−56.78 (16)	O12—C12—C13—O17	27.68 (19)
C6—C1—C2—C21	−174.59 (12)	O11—C12—C13—O17	−153.67 (11)
C1—C2—C21—C22	−68.34 (17)	O12—C12—C13—S14	−89.49 (16)
C3—C2—C21—C22	55.10 (18)	O11—C12—C13—S14	89.16 (12)
C1—C2—C21—C23	167.51 (14)	O17—C13—S14—C15	−33.57 (10)
C3—C2—C21—C23	−69.05 (17)	C12—C13—S14—C15	84.90 (11)
C1—C2—C3—C4	−57.56 (17)	C13—S14—C15—C16	37.16 (10)
C21—C2—C3—C4	175.32 (13)	S14—C15—C16—O17	−33.65 (15)
C2—C3—C4—C5	57.98 (18)	S14—C15—C16—O18	84.12 (12)
C3—C4—C5—C51	−176.96 (15)	O18—C16—O17—C13	−109.17 (13)
C3—C4—C5—C6	−53.74 (19)	C15—C16—O17—C13	9.12 (17)
O11—C1—C6—C5	−177.46 (12)	C12—C13—O17—C16	−97.69 (14)
C2—C1—C6—C5	−57.75 (17)	S14—C13—O17—C16	19.84 (14)
C51—C5—C6—C1	176.82 (14)	O17—C16—O18—C19	−98.38 (14)
C4—C5—C6—C1	53.16 (17)	C15—C16—O18—C19	142.46 (13)
C6—C1—O11—C12	−123.41 (13)	C16—O18—C19—O19	−2.4 (2)
C2—C1—O11—C12	114.39 (14)	C16—O18—C19—C20	177.29 (13)

### Hydrogen-bond geometry (Å, °)

**Table:**

*D*—H···*A*	*D*—H	H···*A*	*D*···*A*	*D*—H···*A*
C13—H13···O12^i^	0.989 (15)	2.261 (15)	3.1563 (18)	150.0 (12)
C15—H15A···S14^ii^	0.961 (16)	3.033 (15)	3.7794 (15)	135.6 (11)
C20—H20B···O19^i^	0.961 (18)	2.524 (18)	3.464 (2)	166.0 (14)

Symmetry codes: (i) *x*+1, *y*, *z*; (ii) *x*−1/2, −*y*+3/2, −*z*+1.

## References

[bb1] Allen, F. H. (2002). *Acta Cryst.* B**58**, 380–388. 10.1107/s0108768102003890 12037359

[bb2] Altomare, A., Cascarano, G., Giacovazzo, C. & Guagliardi, A. (1993). *J. Appl. Cryst* **26**, 343–350.

[bb3] Duax, W. L. & Norton, D. A. (1975). In *Atlas of Steroid Structures*. New York: Plenum.

[bb4] Flack, H. D. (1983). *Acta Cryst.* A**39**, 876–881.

[bb5] Harris, R. K., Yeung, R. R., Lamont, R. B., Lancaster, R. W., Lynn, S. M. & Staniforth, S. E. (1997). *J. Chem. Soc. Perkin Trans. 2*, pp. 2653–2659.

[bb6] Oxford Diffraction (2009). *CrysAlis Pro*. Oxford Diffraction Ltd, Yarnton, England.

[bb7] Pereira, B. G., Vianna-Soares, C. D., Righi, A., Pinheiro, M. V. B., Flores, M. Z. S., Bezerra, E. M., Freire, V. N., Lemos, V., Caetano, E. W. S. & Cavada, B. S. (2007). *J. Pharm. Biomed. Anal.* **43**, 1885–1889. 10.1016/j.jpba.2007.01.014 17303364

[bb8] Sheldrick, G. M. (2008). *Acta Cryst.* A**64**, 112–122. 10.1107/S0108767307043930 18156677

[bb9] Siemens (1989). *Stereochemical Workstation Operation Manual.* Siemens Analytical X-ray Instruments Inc., Madison, Wisconsin, USA.

